# Amelioration of diabetic nephropathy by SGLT2 inhibitors independent of its glucose-lowering effect: A possible role of SGLT2 in mesangial cells

**DOI:** 10.1038/s41598-019-41253-7

**Published:** 2019-03-18

**Authors:** Toshinobu Maki, Sayaka Maeno, Yasutaka Maeda, Mayumi Yamato, Noriyuki Sonoda, Yoshihiro Ogawa, Masanori Wakisaka, Toyoshi Inoguchi

**Affiliations:** 1 Department of Medicine and Bioregulatory Science, Graduate School of Medical Sciences, Kyushu University, Fukuoka, Japan; 2 Minami Masae Clinic, Fukuoka, Japan; 3 Physical Chemistry for Life Science Laboratory, Faculty of Pharmaceutical Sciences, Kyushu University, Fukuoka, Japan; 4 Department of Molecular Endocrinology and Metabolism, Graduate School of Medical and Dental Sciences, Tokyo Medical and Dental University, Tokyo, Japan; 5 Japan Agency for Medical Research and Development, CREST, Tokyo, Japan; 6 Wakisaka Naika (Clinic of Internal Medicine), Fukuoka, Japan; 7 Fukuoka City Health Promotion Support Center, Fukuoka, Japan; 8 Innovation Center for Medical Redox Navigation, Kyushu University, Fukuoka, Japan

## Abstract

Several clinical studies have shown the beneficial effects of sodium-glucose cotransporter 2 (SGLT2) inhibitors on diabetic nephropathy. The underlying mechanisms are not fully understood. We found that administration of canagliflozin at a low dose (0.01 mg/kg/day) did not affect either blood glucose levels or glycosuria, but it improved albuminuria and mesangial expansion in *db/db* mice to a similar extent as at a high dose (3.0 mg/kg/day) that lowered blood glucose levels. This indicated the existence of a tubular SGLT2-independent reno-protective mechanism. Here we focused on the potential role of SGLT2 in mesangial cells (MCs). Western blot analysis revealed the expression of SGLT2 in cultured mouse MCs. Exposure of MCs to high glucose levels for 72 h significantly increased the expression of SGLT2. Canagliflozin or ipragliflozin (both 100 nM) treatment inhibited glucose consumption in the medium under high-glucose conditions but not under normal-glucose conditions. Furthermore, canagliflozin inhibited high-glucose-induced activation of the protein kinase C (PKC)-NAD(P)H oxidase pathway and increases in reactive oxygen species (ROS) production. Thus, the inhibition of mesangial SGLT2 may cause an inhibition of PKC activation and ROS overproduction in diabetic nephropathy, and this may at least in part account for the reno-protective effect of SGLT2 inhibitors.

## Introduction

Diabetic nephropathy is a leading cause of end-stage renal disease (ESRD), and it also contributes to increased cardiovascular morbidity and mortality in type 2 diabetes. Despite increased efforts to optimize renal risk factors by lifestyle and pharmacological interventions, the prevalence of diabetic nephropathy and its related ESRD has not changed sufficiently. Thus, a therapeutic approach targeting its causative mechanisms urgently needs to be established. Sodium-glucose cotransporter 2 (SGLT2) inhibitors are a novel class of anti-hyperglycemic drugs that block the reabsorption of glucose in the kidney, increase urinary glucose excretion, and thus lower blood glucose levels. These medications also exert multiple metabolic effects such as reducing body weight, decreasing blood pressure, and improving lipid profiles^1^. In addition, several clinical studies have suggested the beneficial effects of SGLT2 inhibitors on nephropathy as well as cardiovascular events in patients with type 2 diabetes^2–4^. The potential mechanisms responsible for the reno-protective effect of SGLT2 inhibitors may be multifactorial. In addition to multiple favorable metabolic effects, SGLT2 inhibitors reduce proximal tubular sodium reabsorption, increase sodium delivery to the macular densa, and thus stimulate tubuloglomerular feedback and afferent arteriolar vasomodulation, resulting in decreased glomerular filtration^5,6^. All these effects are mediated by an inhibition of SGLT2 in proximal tubular cells and subsequent increased glycosuria. In this study, we first show the reno-protective effect of SGLT2 inhibitor canagliflozin at a low administered dose that did not significantly affect either blood glucose levels or glycosuria in *db/db* mice. This phenomenon indicates the possible existence of another underlying mechanism that is independent of an inhibitory effect on proximal tubular SGLT2. In addition, we show that SGLT2 exists in renal mesangial cells (MCs), and inhibition of mesangial SGLT2 may lead to an improvement of high-glucose-induced mesangial dysfunctions including protein kinase C (PKC) activation and reactive oxygen species (ROS) overproduction. This may at least in part account for the reno-protective effect of SGLT2 inhibitors.

## Results

### Effect of canagliflozin administration on renal injury in diabetic mice

**Figure 1:**
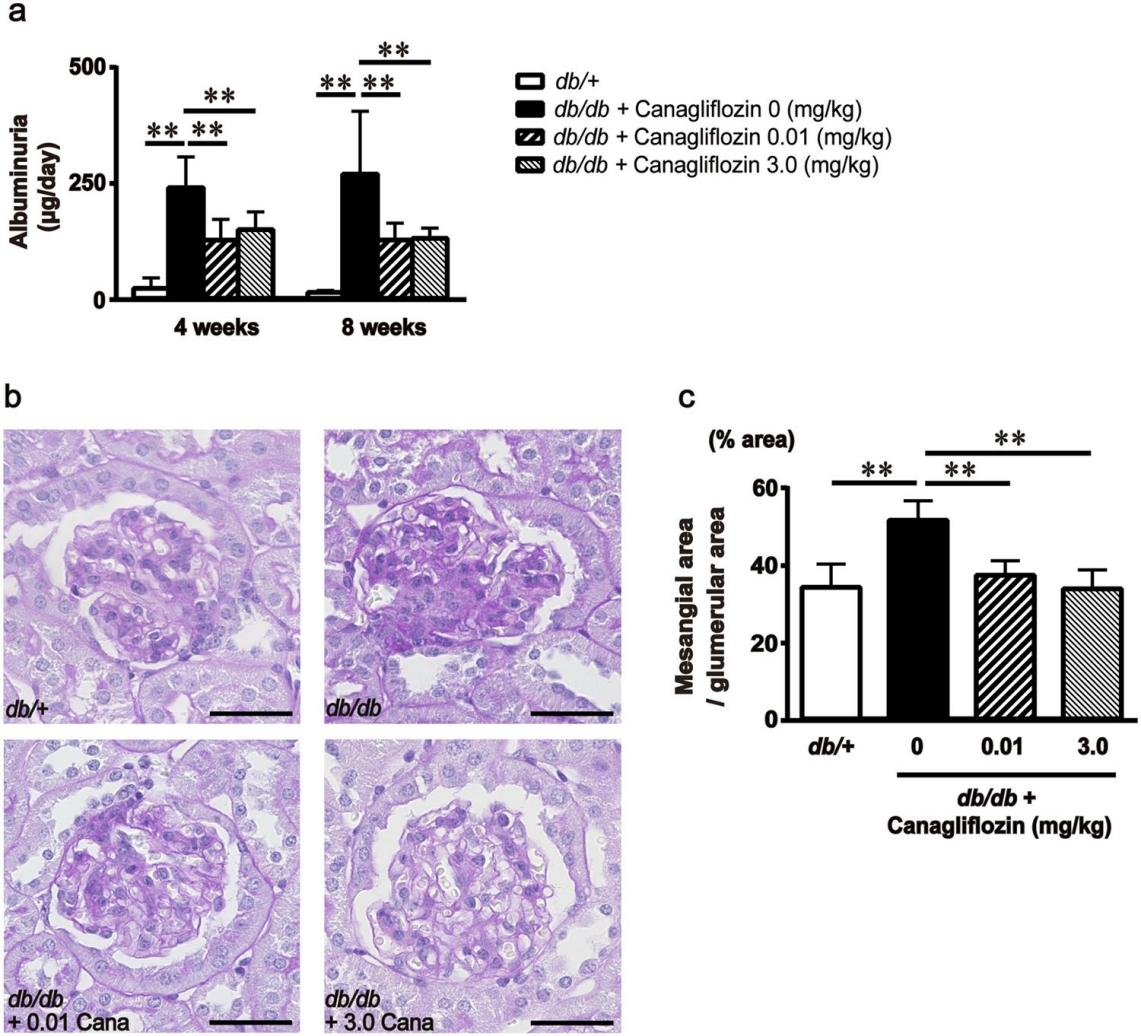
Effect of canagliflozin administration on renal injury in diabetic mice. (**a**) Urinary albumin excretion levels were adjusted according to 24-h urine volume after 4 and 8 weeks’ treatment. Bars represent the means ± SD (n = 7–10). (**b**) Representative photomicrographs showing renal sections stained with periodic acid-Schiff (PAS). (**c**) Percentage of PAS-positive area per glomerular area by quantitative image analysis. Bars represent the means ± SD (n = 5). *P < 0.05, **P < 0.01 vs. non-treated *db/db* mice. Scale bar = 40 μm.

We first examined the dose-dependent effect of oral administration of canagliflozin on blood glucose levels in 12-week-old *db/db* mice (0.001, 0.01, 0.1, 1.0, 3.0 mg/kg/day). After 2 weeks treatment, canagliflozin significantly lowered fasting glucose levels and serum fructosamine levels in a dose-dependent manner, but 0.1 mg/kg/day or less of it did not (Supplementary Fig. S1). This dose-dependency was quite consistent with the previous report^7^. Thus, we compared the reno-protective effect of oral administration of canagliflozin for 8 weeks at a low dose of 0.01 mg/kg/day and a high dose of 3.0 mg/kg/day in *db/db* mice. Characteristics of the experimental mice are summarized in Table 1. A high dose of canagliflozin significantly reduced diastolic blood pressure, serum fructosamine levels, and fasting glucose levels in *db/db* mice, but a low dose of it did not significantly affect body weights, blood pressure, urinary glucose excretion, serum fructosamine levels, or fasting glucose levels. Of particular interest, a low dose of canagliflozin significantly ameliorated albuminuria to a similar extent as a high dose of it did in *db/db* mice (Fig. 1a). In addition, we evaluated the effect of a low dose of canagliflozin on renal mesangial expansion, which is one of the most striking histological characteristics of diabetic nephropathy. Following 8 weeks of treatment, renal mesangial expansion in *db/db* mice was also ameliorated by a low dose of canagliflozin to a similar extent as by high dose of it (Fig. 1b,c).

**Table 1:** Characteristics of the four groups of mice at 8 weeks after treatment. Data are the means ± SD. *P < 0.05 vs. *db/*+ mice, ^†^*P* < 0.05 vs. *db/db* + vehicle.

Characteristic	*db/*+(n = 7)	*db/db* + vehicle(n = 8)	*db/db* + Canagliflozin 0.01 mg/kg(n = 8)	*db/db* + Canagliflozin 3.0 mg/kg(n = 8)
Body weight (g)	27.1 ± 1.5	50.7 ± 3.6*	49.9 ± 3.8*	53.7 ± 2.6*
Food consumption (g/day)	3.5 ± 0.4	5.8 ± 0.9*	5.4 ± 0.9*	5.7 ± 0.3*
Fasting blood glucose levels (mg/dl)	70.1 ± 9.4	354.1 ± 81.6*	331.4 ± 99.6*	146.8 ± 16.7*^†^
Fructosamine (µmol/L)	377 ± 43	451 ± 36*	461 ± 62*	415 ± 23*^†^
Kidney wt/body wt, ×10^−3^	11.5 ± 0.5	8.2 ± 0.8*	7.9 ± 1.0*	7.9 ± 0.6*
Urinary glucose excretion (mg/day)	0.6 ± 0.4	1078.6 ± 590.9*	753.7 ± 458.0*	970.1 ± 252.7*
Systolic blood pressure (mmHg)	121.3 ± 14.4	138.8 ± 6.1*	131.2 ± 12.9	132.0 ± 10.3
Diastolic blood pressure (mmHg)	61.3 ± 17.1	77.1 ± 7.1*	74.4. ± 9.2	68.8 ± 6.8^†^
Mean blood pressure (mmHg)	81.5 ± 15.9	98.0 ± 5.8*	93.2 ± 9.7	90.0 ± 7.7^†^

### Expression of SGLT2 protein in MCs

**Figure 2:**
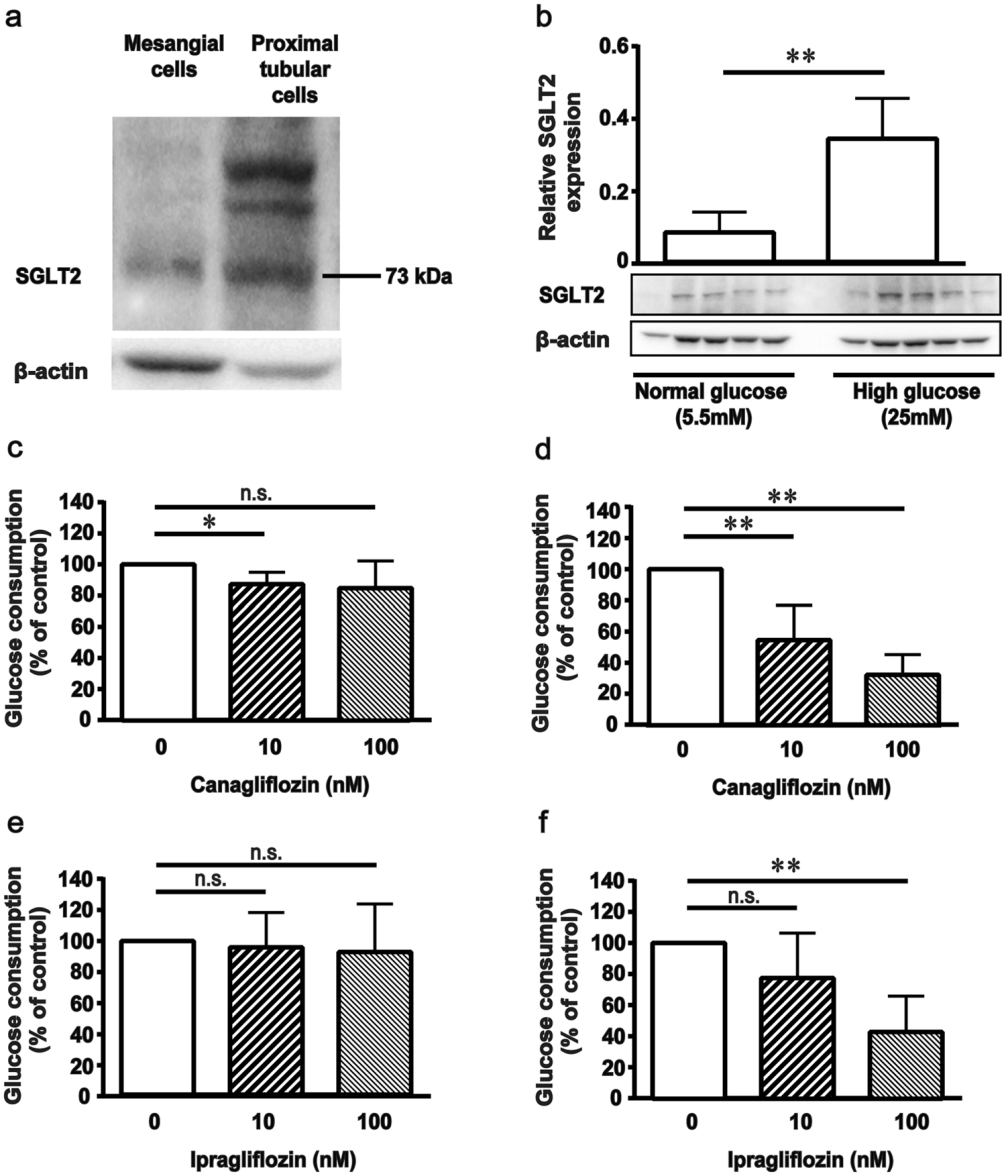
Presence of sodium-glucose cotransporter 2 (SGLT2) and glucose consumption in mouse mesangial cells (MCs). (**a**) Western blotting was performed using anti-SGLT2 and anti-β actin antibodies. (**b**) Induction of protein expression of SGLT2 in mouse MCs under hyperglycemic condition. MCs were plated in 5.5 and 25 mM glucose medium for 3 days. Western blotting was performed using anti-SGLT2 and anti-β actin antibodies. The data represent the means ± SD of five independent experiments. **P < 0.01 vs. normal glucose. Full–length western blots are presented in Supplementary Fig. S2. (**c–f**) Glucose consumption in mouse MCs. MCs were plated in 5.5 and 25 mM glucose medium with or without canagliflozin or ipragliflozin for 3 days. Glucose consumption rates were normalized to untreated cells. The data represent the means ± SD of four independent experiments performed in triplicate. *P < 0.05, **P < 0.01 vs. control, n.s.: non-significant.

This reno-protective effect of a low dose of canagliflozin may indicate the possible existence of mechanism that is independent of its inhibitory effect on proximal tubular SGLT2, since this dose did not affect either blood glucose levels or glycosuria. Here we focused on the impact of canagliflozin on renal MCs. We found *in vitro* expression of SGLT2 in mouse MCs by western blot analysis. Western blot analysis revealed an SGLT2 band in both mouse MCs and renal proximal tubules (Fig. 2a). Furthermore, to evaluate the function of SGLT2 in MCs, we measured the rates of glucose consumption in the medium. Cells were cultured in the medium containing either 5.5 (normal glucose levels) or 25 mM glucose (high glucose levels), with or without canagliflozin for 3 days. When incubated in the medium containing 5.5 mM glucose, glucose consumption in the medium was not significantly affected by canagliflozin (10, 100 nM). In contrast, when incubated in the medium containing 25 mM glucose, glucose consumption was significantly inhibited by canagliflozin in a dose-dependent manner (10, 100 nM) (Fig. 2c,d). These findings were confirmed using ipragliflozin, another SGLT2 inhibitor (Fig. 2e,f), suggesting that glucose entry into MCs was mediated by SGLT2 under high-glucose conditions. Based on these results, we examined whether the expression of SGLT2 was altered by high glucose exposure in MCs. Interestingly, the expression of SGLT2 was significantly increased in response to high-glucose levels compared with normal-glucose levels (Fig. 2b).

### Effect of canagliflozin on oxidative stress in MCs

**Figure 3:**
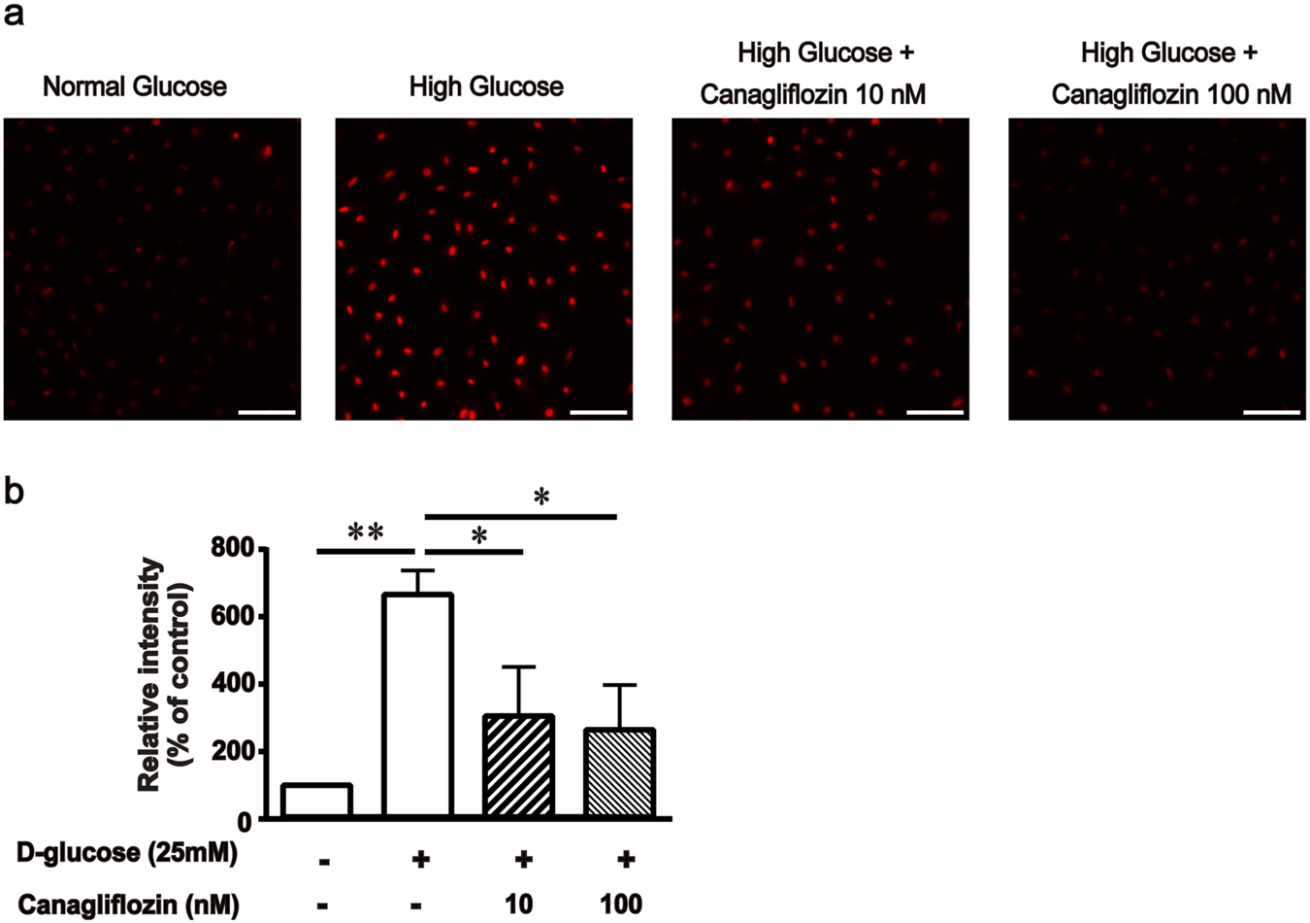
Effect of canagliflozin on superoxide production in mouse mesangial cells (MCs). (**a**) Superoxide production was detected with dihydroethidium (DHE) staining. MCs were treated with or without canagliflozin as indicated in the Methods section. (**b**) Semiquantitative analysis. Results are expressed as the mean percentages of the levels in control ± SD from three independent experiments. *P < 0.05, **P < 0.01 vs. high glucose. Scale bar = 100 μm.

**Figure 4:**
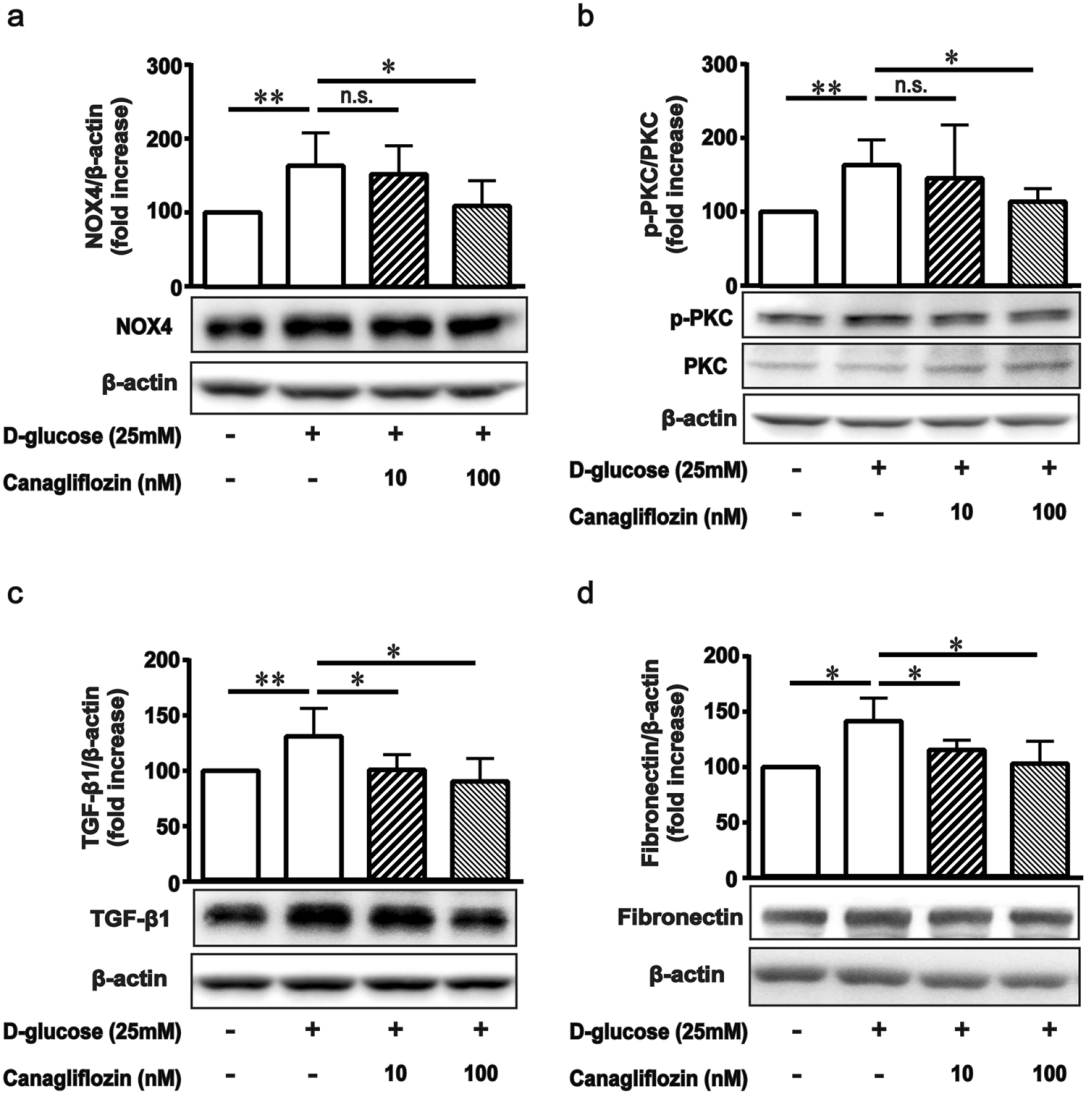
Effect of canagliflozin on expression of proteins that affect oxidative stress and fibrosis in mouse mesangial cells (MCs). (**a–d**) MCs were plated in 5.5 and 25 mM glucose medium with or without canagliflozin for 3 days. Western blotting was performed using anti-NAD(P)H oxidase-4 (NOX4), anti-pan protein kinase C (PKC), anti-pan phospho-PKC, anti-fibronectin, anti-transforming growth factor (TGF)-β1 or anti-β actin antibodies. The data represent the means ± SD of five independent experiments. *P < 0.05, **P < 0.01 vs. high glucose, n.s.: non-significant. Full–length western blots are presented in Supplementary Fig. S3.

We next examined the inhibitory effect of canagliflozin on high-glucose-induced ROS production in MCs. Effect of canagliflozin on intracellular production of ROS was evaluated using dihydroethidium (DHE) staining. Intracellular production of ROS was significantly enhanced under high-glucose conditions compared with normal-glucose conditions, and this was significantly inhibited by canagliflozin treatment in a dose-dependent manner (Fig. 3a,b). In addition, canagliflozin (100 nM) treatment significantly inhibited the high-glucose-induced increased expression of NAD(P)H oxidase-4 (NOX4), which is considered a major source of ROS production in MCs^8,9^, and also inhibited the high-glucose-induced increased phosphorylation of PKC in MCs (Fig. 4a,b). This suggests that canagliflozin normalized ROS overproduction in MCs via inhibition of PKC-NAD(P)H oxidase pathway activation induced by high glucose levels. Furthermore, we found that canagliflozin normalized the expression of transforming growth factor (TGF)-β1, a key cytokine that mediates extracellular matrix accumulation and glomerular expansion in diabetes, and the expression of fibronectin, a predominant matrix protein in glomerular expansion in diabetes (Fig. 4c,d). For SGLT2 expression, canagliflozin did not significantly affect high glucose-induced increase in its expression (Supplementary Fig. 4S).

## Discussion

Several clinical studies have demonstrated the reno-protective effect of SGLT2 inhibitors in patients with nephropathy due to type 2 diabetes^3,4^. The mechanisms responsible for the reno-protective effect of SGLT2 inhibitors are likely to be multifactorial. By increasing distal tubular sodium delivery and stimulating tubuloglomerular feedback, SGLT2 inhibitors increase afferent arteriolar tone and decrease intraglomerular pressure^5^. In addition, they lead to modest decreases in body weight and blood pressure^1^. Decreased sodium reabsorption could affect proximal tubular cell energetics, and thereby it has a favorable effect on other functions of these metabolically active cells. However, it should be noted that these multiple mechanisms are mediated by the inhibition of SGLT2 in proximal tubular cells. In this study, we first examined the dose dependent effect of canagliflozin, and found that the dose of 1.0 or 3.0 mg/kg/day canagliflozin inhibited both fasting blood glucose and serum fructosamine levels in *db/db* mice, but the dose of 0.1 mg/kg/day or less did not significantly affect either of them. This dose-dependency was quite consistent with the previous report showing that canagliflozin at the dose of 0.1 mg/kg/day or less did not significantly affect blood glucose AUC (0–24 h) or urinary glucose AUC (0–24 h) using normal mice^7^. All these results suggest that canagliflozin at dose of 0.1 mg/kg/day or less at least do not have a significant effect on tubular SGLT2. In this study, we showed the reno-protective effect at the dose of 0.01 mg/kg/day to a similar extent as that at 3.0 mg/kg/day, indicating that this effect is likely not to be mediated by the inhibition of tubular SGLT2.

Glomerular hyperfiltration is explained by the glomerular hemodynamics or tubuloglomerular feedback hypothesis, while MCs also play an important role in the maintenance and regulation of microvascular blood flow in glomerulus^10^. Thus, impaired contractility of MCs induced by a high-glucose or diabetes state has been thought to cause glomerular hyperfiltration^11,12^. In addition, overproduction of extracellular matrices by MCs may also contribute to basement membrane thickening and mesangial expansion^13^, which are characteristic changes of diabetic nephropathy.

We and others have previously reported that high glucose levels stimulate *de novo* synthesis of diacylglycerol (DAG), which depends on excess glucose entry into the cells through glucose transporter, followed by PKC activation^14,15^ and subsequent NAD(P)H oxidase-driven ROS overproduction^16,17^ in various vascular cells. In MCs, DAG-PKC pathway activation induced the increased expression of NOX4, subsequent ROS overproduction^18^, and increased expression of TGF- β and fibronectin^8^. Wakisaka *et al*. previously reported that MCs exhibit not only glucose transporter 1 (GLUT1) but also sodium-dependent and phlorizin-sensitive glucose transporter. It should be noted that the Km value of this sodium-dependent transporter for D-glucose was 1.93 mM, which was consistent with that of SGLT2^19^. Recently, they showed the presence of SGLT protein and mRNA in rat MCs corresponding to SGLT2^20^. In our study, we confirmed the presence of SGLT2 in mouse MCs and found that SGLT2 inhibitors (canagliflozin, ipragliflozin) inhibited glucose consumption in the medium under a high-glucose condition but not under a normal-glucose condition. This difference between high-glucose and normal-glucose conditions may be explained by the phenomenon that SGLT2 expression is increased in response to high glucose exposure. The mechanism underlying high-glucose-induced increased SGLT2 expression remained to be clarified in future studies. Subsequently, canagliflozin (100 nM) treatment inhibited a high-glucose-induced increase in ROS production via activation of PKC-NAD(P)H oxidase pathway and increases in expression of TGF-β1 and fibronectin, which are associated with mesangial expansion in diabetic kidneys.

It is reported that canagliflozin has relatively weak selectivity for SGLT2 over SGLT1 (>160-fold) compared with other SGLT2 inhibitors such as dapagliflozin (>1200-fold), empagliflozin (>2500-fold)^1^. Canagliflozin inhibits human SGLT1 or human SGLT2-mediated transport of alpha-methyl-D-glucopyranoside with an IC_50_ of 663 or 4.2 nM, respectively^21^. Within the concentration range of 10–100 nM in the present study, canagliflozin can inhibit SGLT2 but not SGLT1, suggesting that the inhibitory effect of canagliflozin on glucose entry into MCs was thought to be mediated by SGLT2 but not SGLT1. Since ipragliflozin has a higher selectivity for SGLT2 over SGLT1 than does canagliflozin^1^, the effect of ipragliflozin in the present study was also thought to be mediated by SGLT2.

However, it is still unknown how a low dose (0.01 mg/kg/day) of canagliflozin produced a similar reno-protective effect to the higher dose of it in a mouse study. It is well established that canagliflozin acts on tubular SGLT2 from the urinal side^22^. It is possible that the low dose did not produce an effective concentration in urine (thus did not have a glucose lowering effect), but might produce an effective concentration in plasma or local renal tissue enough to inhibit mesangial SGLT2, because the excretion of canagliflozin in urine is only <1% of the administered dose^23^. In addition, several SGLT2 inhibitors including canagliflozin were reported to be highly distributed and retained long in the kidney comparing to other organs^7^. Therefore, one possible explanation is that the inhibitory effect on mesangial SGLT2 might reach a plateau at this low dose, and thus it might be the same even at the higher dose. The effectiveness of low dose might be applicable to other SGLT2 inhibitors in varying degrees. However, this hypothesis should be evaluated in further studies.

In conclusion, our results showed that a low dose of SGLT2 inhibitor canagliflozin has a reno-protective effect independent of its glucose-lowering effect. This pleiotropic effect may be at least in part attributed to an inhibition of mesangial SGLT2, followed by an inhibition of high-glucose-induced DAG-PKC activation and ROS overproduction.

## Methods

### Animals

Ten-week-old male BKS.Cg-*Dock 7* ^*m*+/+^*Lepr*^*db*^/J (*db/db*) mice and their age-matched non-diabetic lean control littermates (*db/*+) were purchased from Clea Japan Inc. (Tokyo, Japan). All mice were bred under pathogen-free conditions at the Center of Biomedical Research, Research Center for Human Disease Modeling, Graduate School of Medical Sciences, Kyushu University (Fukuoka, Japan). The animals had free access to tap water and standard chow (Clea Japan Inc.) containing 50.1% carbohydrates, 25.1% proteins, 7.1% minerals, 4.5% fat, and 4.3% cellulose. Canagliflozin (Mitsubishi Tanabe Pharma Co., Tokyo, Japan) was suspended in 0.5% methylcellulose solution and administered via oral gavage. To examine the effect of canagliflozin administration on blood glucose levels in diabetic mice, the vehicle or various doses of canagliflozin (0.001–3.0 mg/kg/day) was administered orally to diabetic mice for 2 weeks at the age of 12 weeks. To examine the effect of canagliflozin administration on renal injury in diabetic mice, canagliflozin (0.01 or 3.0 mg/kg/day) was administrated to *db/db* mice for 8 weeks starting at the age of 12 weeks. Control *db/db* mice and control *db/*+ mice received 0.5% methylcellulose solution alone for the same period. Blood samples were obtained at each sampling point. A 24-h urine sample was collected using metabolic cages (one mouse per cage) for the last 2 days of the 4-week or 8-week treatment. At the end of the treatment, all mice were anesthetized with isoflurane and a blood sample obtained by cardiac puncture for the determination of fructosamine, and then mice were killed by exsanguination. The kidney was immediately excised and stored in formalin for the following experiments. All animals’ procedures were reviewed and approved by the Ethics Committee of Animal Experiments, Graduate School of Medical Sciences, Kyushu University. All methods were performed in accordance with the relevant guidelines and regulations. Every effort was made to minimize the number of animals used and their suffering.

### Blood and urine analysis

Plasma glucose, fructosamine, and urinary albumin concentrations were determined as described previously^24^. The results of these urinary excretions over 24 hours were calculated as analyte concentration × urinary volume corrected to 24 hours. Blood pressure was measured using a tail cuff method (MK-2000; Muromachi Kikai, Tokyo, Japan). All mice were acclimated to the restraint and tail cuff inflation for 3 days before the beginning of the experiments.

### Morphological study

Fixed kidneys were embedded in paraffin, sectioned (3 µm) and stained with periodic acid-Schiff (PAS). The mesangial matrix was identified by the presence of PAS-positive and nuclei-free areas in the mesangium. The obtained images were measured and adjusted by glomerular area in every glomerulus using a software package (Keyence, Osaka, Japan). An average of 50 glomeruli per mouse were examined and averaged for morphometric analysis.

### Isolation of glomeruli

Isolation of glomeruli was performed using Dynabeads M-450 tosylactivated and Magnetic Particle Concentrator (Dynal AS, Oslo, Norway) as previously described^24,25^.

### Materials

Anti-NOX4, anti-PKC, anti-SGLT2, anti-fibronectin antibodies were purchased from Abcam (Cambridge, MA, USA). Anti-pan phospho-PKC, anti-TGF-β antibodies were purchased from Cell Signaling Technology (Danvers, MA, USA). Horseradish peroxidase (HRP)-conjugated donkey anti-rabbit immunoglobulin G (IgG) secondary antibody was obtained from Amersham Biosciences (GE Healthcare Bio-Sciences Corp., Piscataway, NJ, USA). Anti-β-actin HRP-conjugated antibody was obtained from Santa Cruz (Dallas, TX, USA). All other chemicals and reagents were purchased from standard suppliers unless otherwise mentioned.

### Cell culture

MCs were isolated from the kidneys of *db/*+ *mice* (12 weeks old). Cells were cultured on gelatin-coated culture plates (Iwaki Glass, Tokyo, Japan) in Dulbecco’s modified Eagle’s medium (DMEM) supplemented with 20% fetal bovine serum (FBS; GIBCO BRL, Grand Island, NY), penicillin (100 U/ml), and streptomycin (100 μg/ml) (Sigma-Aldrich, St. Louis, MO, USA). MCs at passages 3–8 were used for the following experiments after reaching greater than 80–90% confluence because the SGLT expression in MCs has been reported to disappear after the 12th passage^19^. Renal proximal tubular cells from the same mouse were prepared as previously described^26^. For the experiments, cells are allowed to reach 60–70% confluence in the multiwell plates, and then the medium was changed to 0.5% FBS DMEM containing either 5.5 mM glucose plus 19.5 mM mannitol (to ensure equal osmolarity) or 25 mM glucose with or without canagliflozin or ipragliflozin (Astellas Pharma Inc., Tokyo, Japan), and incubated for 72 h. Canagliflozin or ipragliflozin was dissolved in dimethyl sulfoxide (DMSO) to reach the desired final concentration prior to use, and DMSO was used as the control. The medium was changed daily to maintain the desired glucose and canagliflozin levels. All experiments were performed in triplicates and repeated at least three times.

### Glucose consumption

MCs were seeded in 24-well plates at a density of 1 × 10^4^ cells/well and incubated until 70–80% confluence. Aliquots of the culture media were withdrawn at time 0 and 72 h of incubation and the glucose concentration was measured by glucose oxidase method. Glucose consumption was calculated by the difference between the glucose content of aliquots withdrawn at time 0 and after 72 h of incubation.

### ROS detection

The effect of canagliflozin on intracellular production of superoxide was evaluated in mouse MCs using DHE (Invitrogen, Carlsbad, CA, USA) staining as previously described^27^. The averages of fluorescence intensity values from 40 to 50 cells of three different examinations were calculated. The fluorescence intensity was quantified using BZ image analysis software (Keyence).

### Western blot analysis

After 72 h of incubation with or without canagliflozin in the presence of 5.5 mM or 25 mM glucose, MCs were collected and sonicated in 400 µl of solubilizing solution containing 9 M urea, 2% Triton X-100, and 1% dithiothreitol. Then, 100 µl of 10% lithium dodecyl sulfate was added to the solubilized membrane fraction and sonicated again. Subsequently, proteins were separated discontinuously on 7.5% sodium dodecyl sulfate polyacrylamide gels and transferred to PVDF membranes (Bio-Rad, Hercules, CA, USA). After blocking nonspecific binding, the membranes were incubated overnight at 4 °C with rabbit anti-NOX4 (1:1000; Abcam), anti-PKC (1:250; Abcam), anti-pan phospho-PKC (1:1000; Cell Signaling Technology), anti-SGLT2 (1:500; Abcam), anti-TGF-β (1:500; Cell Signaling Technology), or anti-fibronectin (1:5000; Abcam) antibodies, followed by HRP-conjugated donkey anti-rabbit IgG antibody (1:10,000; Amersham) as a secondary antibody. The membranes were also incubated with goat anti-β-actin HRP-conjugated antibody (1:5000; Santa Cruz) to ensure equal protein loading of the lanes. We used the ECL Plus system (Amersham) to detect antibody binding.

### Statistical analysis

All data are expressed as the means ± SD. Statistical analysis was performed with Student’s t-test or one-way analysis of variance (ANOVA) with Fisher’s protected least significant difference (PLSD) test. A *P*-value of *P* < 0.05 was considered statistically significant.

## Supplementary information

Supplementary Information
