# Supplementary material for: Amelioration of diabetic nephropathy by SGLT2 inhibitors independent of its glucose-lowering effect: A possible role of SGLT2 in mesangial cells
Source: Sci Rep. 2019 Mar 18;9:4703. doi: 10.1038/s41598-019-41253-7 (PMC6423112; doi:10.1038/s41598-019-41253-7)

**Supplementary Information**

**Amelioration of diabetic nephropathy by SGLT2 inhibitors independent of its glucose-lowering effect: A possible role of SGLT2 in mesangial cells**

Toshinobu Maki^1^, Sayaka Maeno^1^, Yasutaka Maeda^2^, Mayumi Yamato^3^, Noriyuki Sonoda^1^, Yoshihiro Ogawa^1, 4, 5^, Masanori Wakisaka^6^ & Toyoshi Inoguchi^7, 8,*^

^1^ Department of Medicine and Bioregulatory Science, Graduate School of Medical Sciences, Kyushu University, Fukuoka, Japan; ^2^ Minami Masae Clinic, Fukuoka, Japan; ^3^ Physical Chemistry for Life Science Laboratory, Faculty of Pharmaceutical Sciences, Kyushu University, Fukuoka, Japan; ^4^ Department of Molecular Endocrinology and Metabolism, Graduate School of Medical and Dental Sciences, Tokyo Medical and Dental University, Tokyo, Japan; ^5^ Japan Agency for Medical Research and Development, CREST, Tokyo, Japan; ^6^ Wakisaka Naika (Clinic of Internal Medicine), Fukuoka, Japan; ^7^ Fukuoka City Health Promotion Support Center, Fukuoka, Japan; ^8^ Innovation Center for Medical Redox Navigation, Kyushu University, Fukuoka, Japan.

**Corresponding Author**

Toyoshi Inoguchi, M.D., Ph.D.

Fukuoka City Health Promotion Support Center

2-5-1 Maizuru, Chuou-ku, Fukuoka City 810-0073, Japan

Fax: +81-92-751-2572 / Tel: +81-92-751-7778

E-mail: toyoshi.inoguchi@gmail.com

**Supplementary Figure S1.** Effect of canagliflozin administration on (**a**) fasting blood glucose levels, (**b**) serum fructosamine levels in diabetic mice. Canagliflozin (0.001, 0.01, 0.1, 1.0, 3.0 mg/kg/day) was given for 2 weeks starting at age of 12 weeks. Bars represent the means ± SD (n = 4–5). * *P* < 0.05, ** *P* < 0.01 vs. non-treated *db/db* mice.


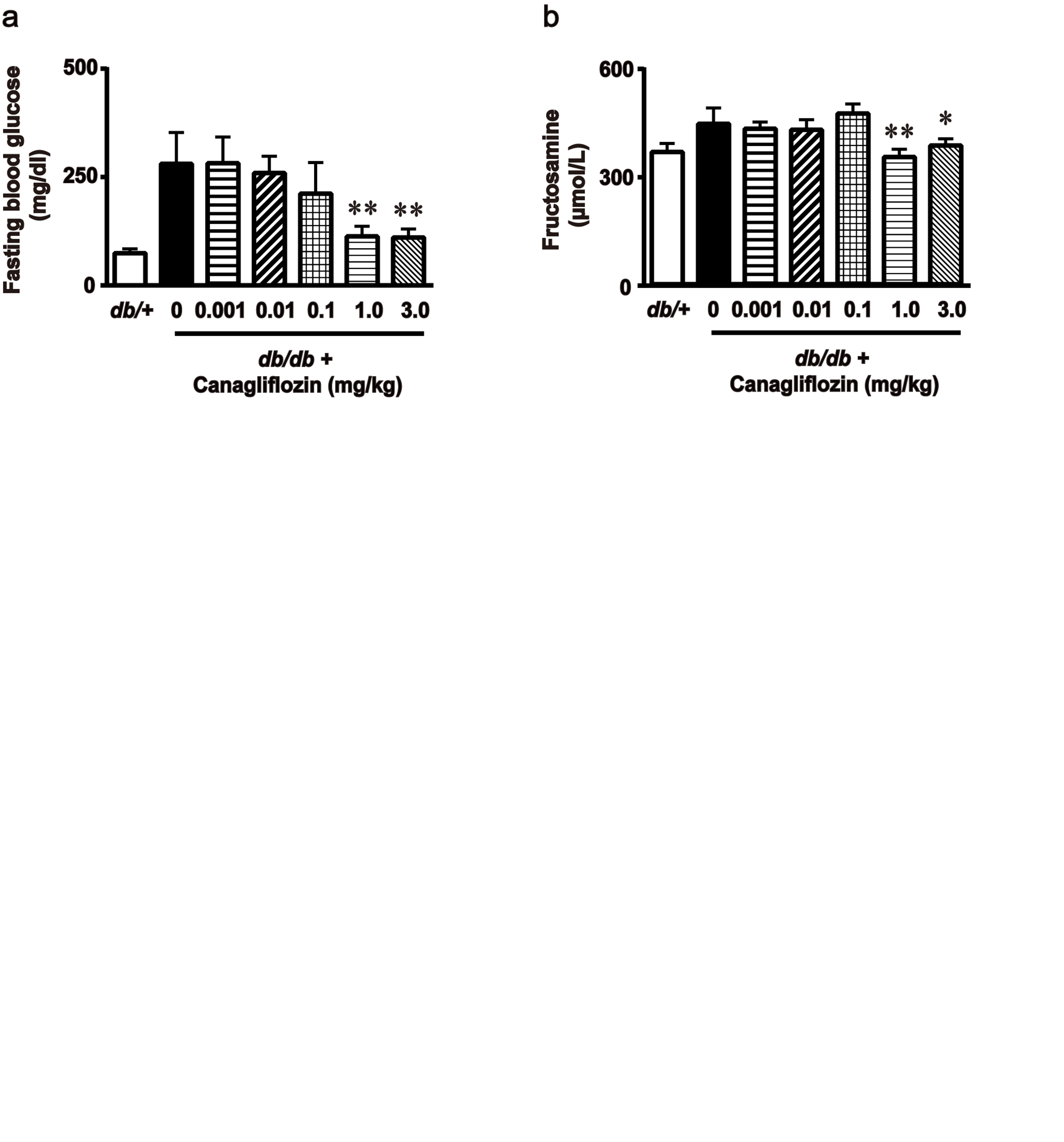


**Supplementary Figure S2.** Full-length blots of Figure 2


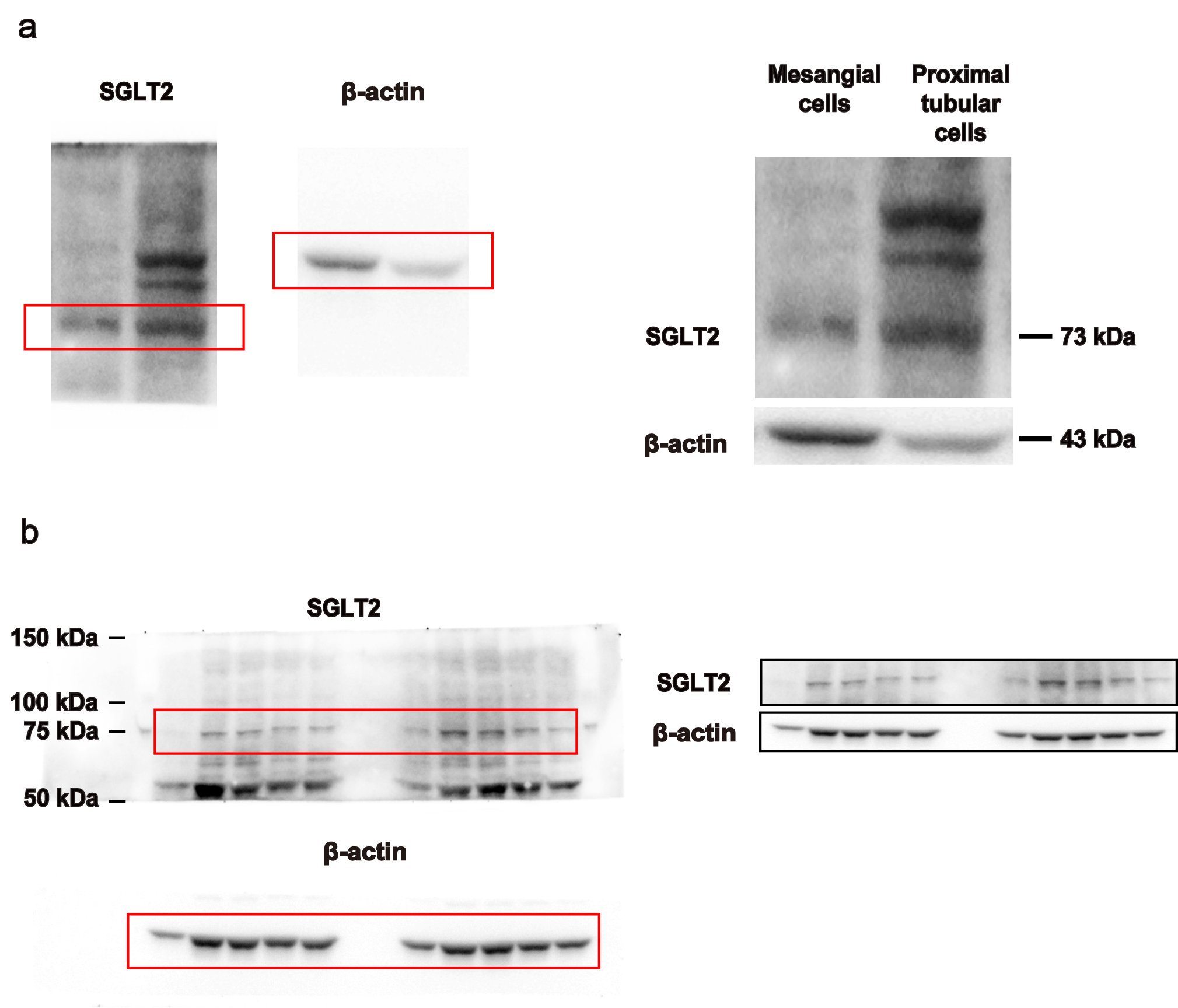


**Supplementary Figure S3.** Full-length blots of Figure 4


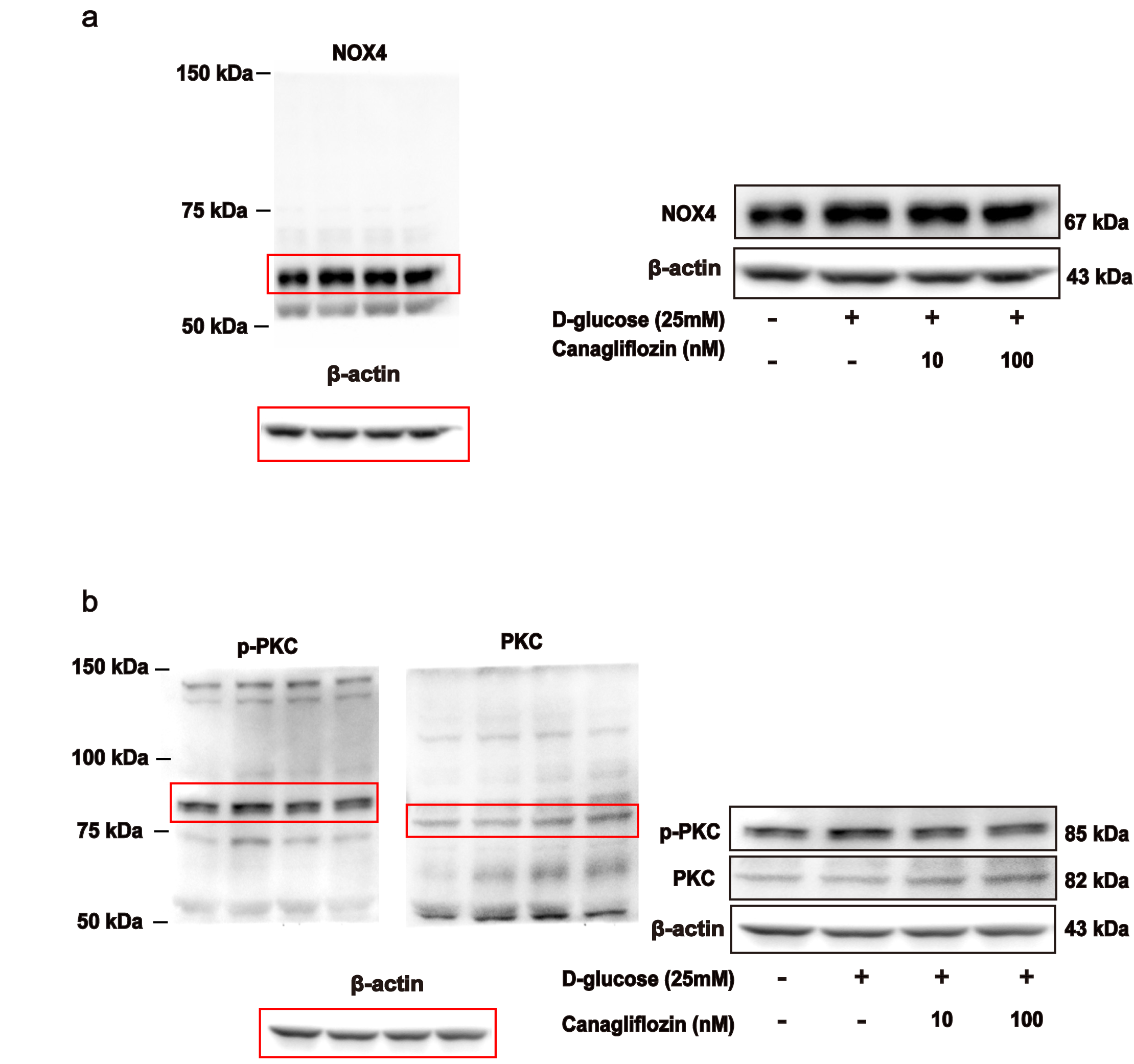


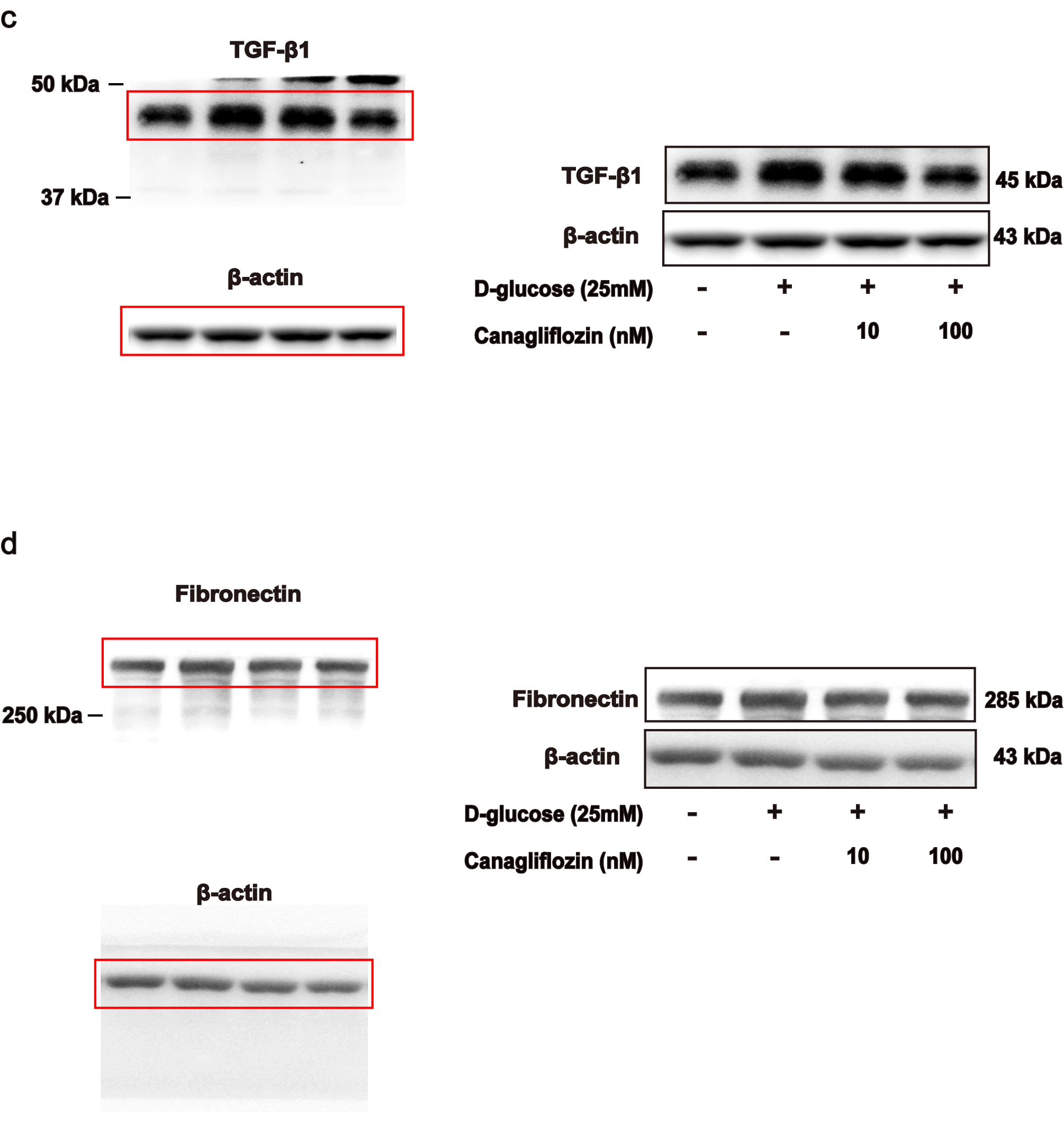


**Supplementary Figure S4.** Induction of protein expression of sodium-glucose cotransporter 2 (SGLT2) in mouse mesangial cells (MCs) under hyperglycemic condition. (**a**) MCs were plated in 5.5 and 25 mM glucose medium with or without canagliflozin for 3 days. Western blotting was performed using anti-SGLT2 and anti-β actin antibodies. The data represent the means ± SD of five independent experiments. ** *P* < 0.01 vs. high glucose, n.s.: non-significant. (**b**) Full–length western blots.


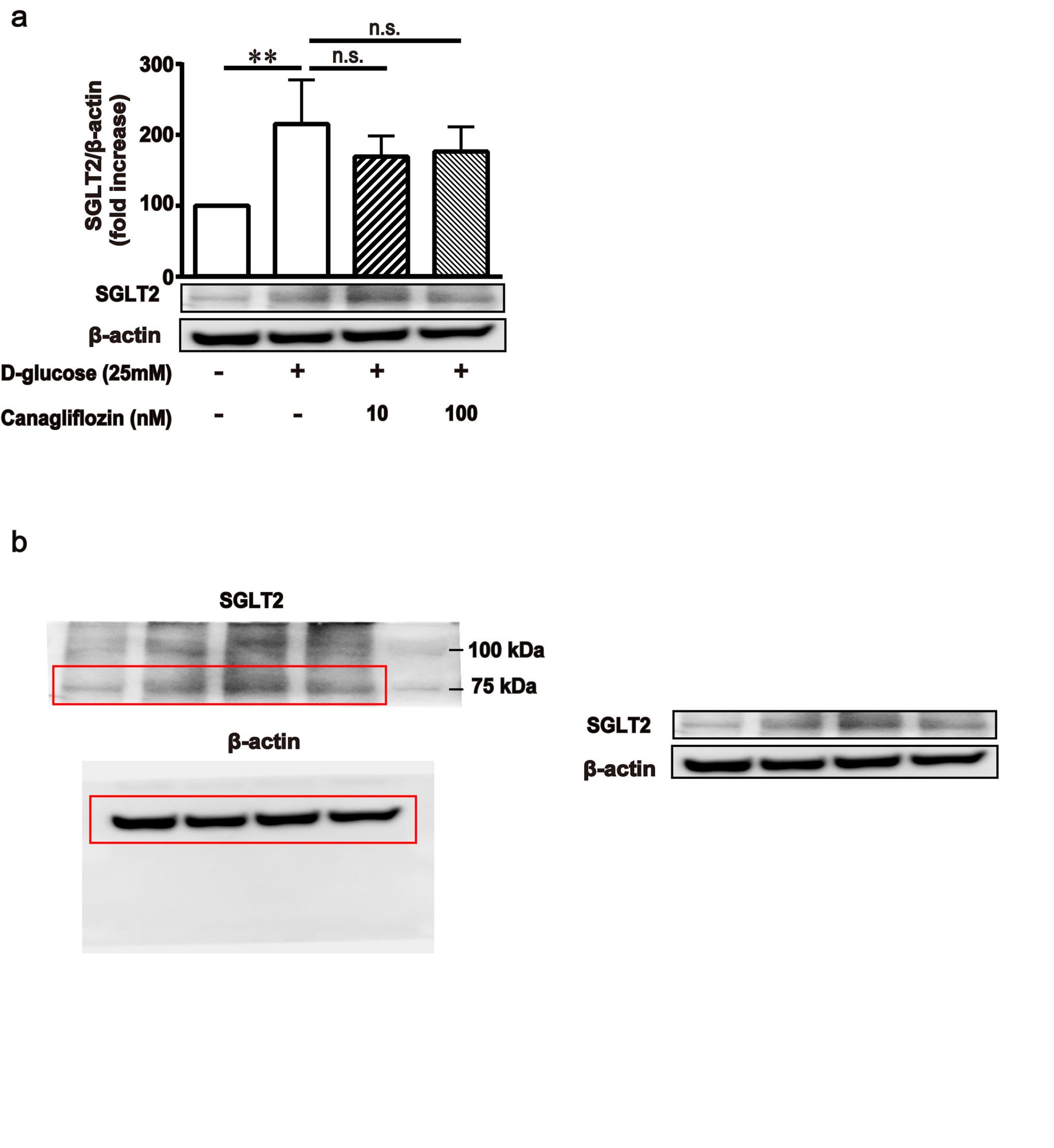

Supplement: Supplementary file 1 — Supplementary Information [file 41598_2019_41253_MOESM1_ESM.doc]
